# Supplementary material for: Characterization of Papillomatous Lesions and Genetic Diversity of Bovine Papillomavirus from the Amazon Region
Source: Viruses. 2025 May 16;17(5):719. doi: 10.3390/v17050719 (PMC12115847; doi:10.3390/v17050719)
Supplement: Supplementary file 1 [file viruses-17-00719-s001.zip › Supplementary materials Table S1.pdf]

Table 1: Animal data, morphology, anatomical location and histological classification of lesions, types and putative new types (PNT) of BPVs found in the study.

BPV type or PNT	Farm	Geographical location	Gender	Age	Anatomic sites of lesions	Morphology	Histopathology	Sample identification	GenBank acession number
**BPV2**	II	12º7′51.24′′S<br>63º37′26.87′′W	Female	12 months	Dewlap	Pedunculated	Fibropapilloma	BRA04RO21	OP682842
	V	12º2′40.39′′S<br>63º50′38.73′′W	Female	12 months	Head	Flat	Fibropapilloma	BRA07RO21	OP682845
	VII	12º7′49.92′′S<br>63º46′47.53′′W	Female	18 months	Abdomen	Flat	Fibropapilloma	BRA11RO21b	OP682846
			Female	18 months	Head	Cauliflower	Fibropapilloma	BRA12RO21	OP682847
			Male	9 months	Tail	Cauliflower	Fibropapilloma	BRA13RO21b	OP682848
	IX	12º4′51.31′′S<br>63º52′14.56′′W	Female	8 years	Back	Flat	NA	BRA15RO21	OP682850
	XII	12º6′10.63′′S<br>63º41′4.16′′W	Female	18 months	Ear	Pedunculated	Fibropapilloma	BRA22RO21a	OP682854
					Head	Cauliflower		BRA22RO21b	OP682855
			Female	18 months	Neck	Cauliflower	Fibropapilloma	BRA23RO21	OP682856
	XIII	12º7′46.00′′S<br>63º39′45.16′′W	Female	18 months	Back	Flat	NA	BRA25RO21b	OP682858
			Female	18 months	Head	Cauliflower	Fibropapilloma	BRA27RO21	OP682861
			Female	18 months	Thorax	Flat	Fibropapilloma	BRA28RO21	OP682862
			Female	18 months	Thorax	Flat	Fibropapilloma	BRA29RO21	OP682863
			Female	18 months	Nostril	Flat	Squamous papilloma	BRA30RO21a	OP682865
					Eye	Cauliflower	Fibropapilloma	BRA30RO21b	OP682864
			Female	18 months	Neck	Flat	Squamous papilloma	BRA31RO21b	OP682866
			Female	18 months	Head	Cauliflower	Fibropapilloma	BRA32RO21a	OP682868
					Back	Flat		BRA32RO21b	OP682867
			Female	16 months	Back	Flat	NA	BRA34RO21	OP682869
			Female	18 months	Thorax	Cauliflower	Fibropapilloma	BRA35RO21	OP682870
	XIV	12º6′13.39′′S<br>63º45′6.98′′W	Male	18 months	Neck	Flat	Fibropapilloma	BRA39RO21a	OP682873
						Cauliflower		BRA39RO21b	OP682872
			Male	14 months	Neck	Cauliflower	Fibropapilloma	BRA40RO21	OP682874
**BPV4**	XIV	12º6′13.39′′S<br>63º45′6.98′′W	Female	2 years	Neck	Flat	Squamous papilloma	BRA41RO21	OP682875
**BPV5**	III	12º5′2.02′′S<br>63º43′37.96′′W	Female	18 years	Dewlap	Flat	Squamous papilloma	BRA05RO21	OP682843
**BPV12**	XIV	12º6′13.39′′S<br>63º45′6.98′′W	Female	2 years	Thorax	Flat	Squamous papilloma	BRA42RO21	OP682876
**BPV13**	I	12º7′21.23′′S<br>63º43′38.66′′W	Male	8 months	Head	Cauliflower	Fibropapilloma	BRA01RO21	OP682841
			Female	8 months	Nostril	Cauliflower	Squamous papilloma	BRA44RO21	OP682878

	VIII	12º3’39.41’’S 63º48’36.98’’W	Female	16 months	Neck	Cauliflower	Fibropapilloma	BRA14RO21	OP682849
	X	12º6’42.75’’S 63º43’40.24’’W	Male	8 months	Thorax	Cauliflower	Squamous papilloma	BRA18RO21	OP682852
	XI	12º8’33.83’’S 63º41’14.91’’W	Female	4 years	Back	Cauliflower	Fibropapilloma	BRA20RO21a	OP682853
	XIII	12º7’46.00’’S 63º39’45.16’’W	Female	18 months	Eye	Cauliflower	Fibropapilloma	BRA24RO21	OP682857
			Female	18 months	Ear	Cauliflower	Fibropapilloma	BRA25RO21a	OP682859
**BPV14**	XIV	12º6’13.39’’S 63º45’6.98’’W	Male	16 months	Neck	Flat	Squamous papilloma	BRA38RO21	OP682871
	XV	12º7’29.55’’S 63º39’9.43’’W	Female	20 months	Neck	Flat	Fibropapilloma	BRA43RO21	OP682877
**BPV15**	IV	12º7’33.58’’S 63º46’57.98’’W	Female	4 years	Back	Flat	Squamous papilloma	BRA06RO21	OP682844
**PNT**	X	12º6’42.75’’S 63º43’40.24’’W	Female	5 years	Tail	Flat	Squamous papilloma	BRA17RO21	OP682851
	XIII	12º7’46.00’’S 63º39’45.16’’W	Female	16 months	Back	Flat	NA	BRA26RO21	OP682860
			Female	18 months	Back	Flat	NA	BRA33RO21	OQ550039

(NA) Sample not analyzed because there was not enough tissue. (PNT) Putative new type of the BPV identified in the present study.
