# Supplementary material for: Characterization of Papillomatous Lesions and Genetic Diversity of Bovine Papillomavirus from the Amazon Region
Source: Viruses. 2025 May 16;17(5):719. doi: 10.3390/v17050719 (PMC12115847; doi:10.3390/v17050719)
Supplement: Supplementary file 1 [file viruses-17-00719-s001.zip › Supplementary materials Table S2.pdf]

Table 2: Clinicopathological characteristics of warts and types of BPV identified in the study (n = 34).

Viral type		Anatomic site			Morphology of the lesions			Microscopy	
		Head, neck or dewlap	Thorax, back or abdomen	Udder, groin or tail	Flat	Cauliflower	Pedunculated	Fibropapilloma	Squamous papilloma
**BPV2**	Positive (%)	14 (41.2)	5 (14.7)	1 (2.9)	8 (23.5)	10 (29.4)	2 (5.9)	17 (50.0)	3 (8.8)
	Negative (%)	9 (26.5)	4 (11.8)	1 (2.9)	7 (20.6)	7 (20.6)	0 (0.0)	6 (17.7)	8 (23.5)
	p	**1.000**			**0.641**			**0.023**	
**BPV4**	Positive (%)	1 (2.9)	0 (0.0)	0 (0.0)	1 (2.9)	0 (0.0)	0 (0.0)	0 (0.0)	1 (2.9)
	Negative (%)	22 (64.7)	9 (26.5)	2 (5.9)	14 (41.2)	17 (50.0)	2 (5.9)	23 (67.7)	10 (29.4)
	p	**1.000**			**0.500**			**0.324**	
**BPV5**	Positive (%)	1 (2.9)	0 (0.0)	0 (0.0)	1 (2.9)	0 (0.0)	0 (0.0)	0 (0.0)	1 (2.9)
	Negative (%)	22 (64.7)	9 (26.5)	2 (5.9)	14 (41.2)	17 (50.0)	2 (5.9)	23 (67.7)	10 (29.4)
	p	**1.000**			**0.500**			**0.324**	
**BPV12**	Positive (%)	0 (0.0)	1 (2.9)	0 (0.0)	1 (2.9)	0 (0.0)	0 (0.0)	0 (0.0)	1 (2.9)
	Negative (%)	23 (67.7)	8 (23.5)	2 (5.9)	14 (41.2)	17 (50.0)	2 (5.9)	23 (67.7)	10 (29.4)
	p	**0.549**			**0.303**			**0.098**	
**BPV13**	Positive (%)	5 (14.7)	2 (5.9)	0 (0.0)	0 (0.0)	7 (20.6)	0 (0.0)	5 (14.7)	2 (5.9)
	Negative (%)	18 (52.9)	7 (20.6)	2 (5.9)	15 (44.1)	10 (29.4)	2 (5.9)	18 (52.9)	9 (26.5)
	p	**1.000**			**0.008**			**1.000**	
**BPV14** *	Positive (%)	2 (5.9)	0 (0.0)	0 (0.0)	2 (5.9)	0 (0.0)	0 (0.0)	1 (2.95)	1 (2.95)
	Negative (%)	21 (61.7)	9 (26.5)	2 (5.9)	13 (38.2)	17 (50.0)	2 (5.9)	22 (64.7)	10 (29.4)
	p	**1.000**			**0.303**			**1.000**	
**BPV15**	Positive (%)	0 (0.0)	1 (2.9)	0 (0.0)	1 (2.9)	0 (0.0)	0 (0.0)	0 (0.0)	1 (2.9)
	Negative (%)	23 (67.7)	8 (23.5)	2 (5.9)	14 (41.2)	17 (50.0)	2 (5,9)	23 (67.7)	10 (29.4)
	p	**0.324**			**0.500**			**0.324**	
**PNT**	Positive (%)	0 (0.0)	0 (0.0)	1 (2.9)	1 (2.9)	0 (0.0)	0 (0.0)	0 (0.0)	1 (2.9)
	Negative (%)	23 (67.7)	9 (26.5)	1 (2.9)	14 (41.2)	17 (50.0)	2 (5.9)	23 (67.7)	10 (29.4)
	p	**0.059**			**0.500**			**0.324**	

p value calculated using Fisher's exact test for 2x2 comparisons and Monte Carlo simulation used for 2x3 comparisons. $p < 0.05$ considered significant.

* Putative new viral subtype.

(PNT) Putative new viral type.
