# Supplementary figures and images for: Characterization of Papillomatous Lesions and Genetic Diversity of Bovine Papillomavirus from the Amazon Region
Source: Viruses. 2025 May 16;17(5):719. doi: 10.3390/v17050719 (PMC12115847; doi:10.3390/v17050719)

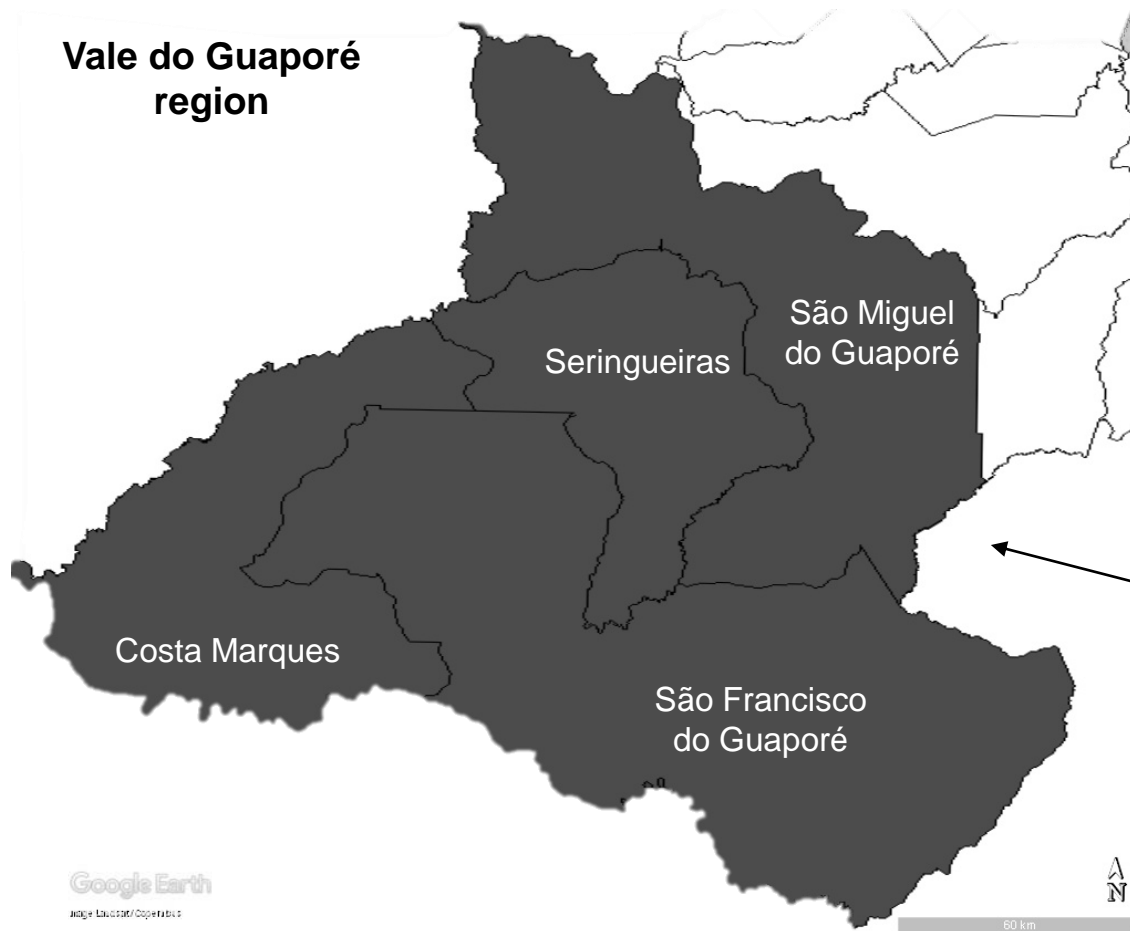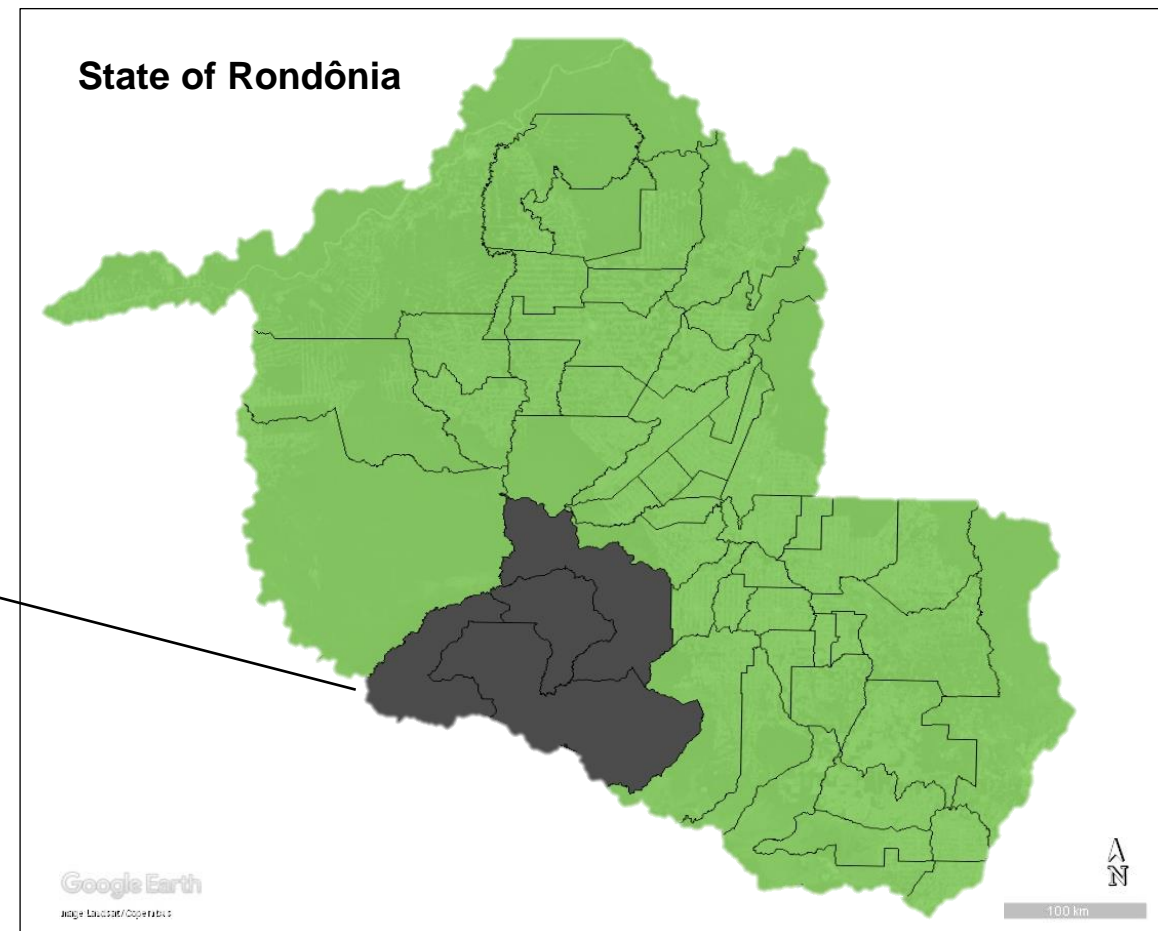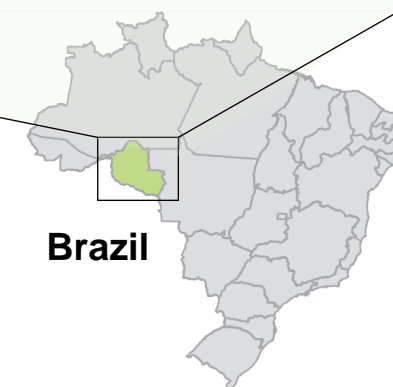

Supplement: Supplementary file 1 [file viruses-17-00719-s001.zip › Supplementary materials Figure S1.pdf]

a

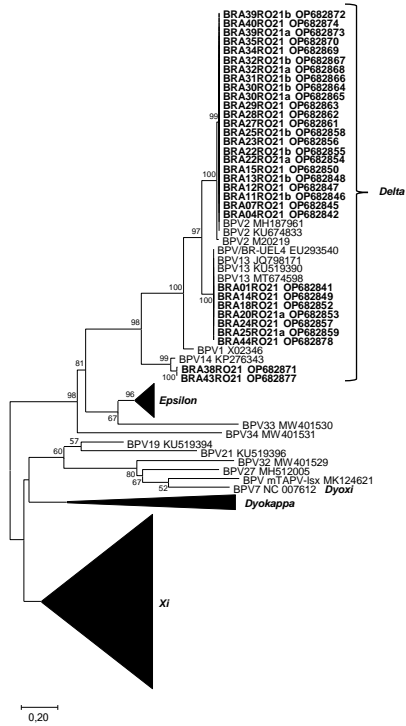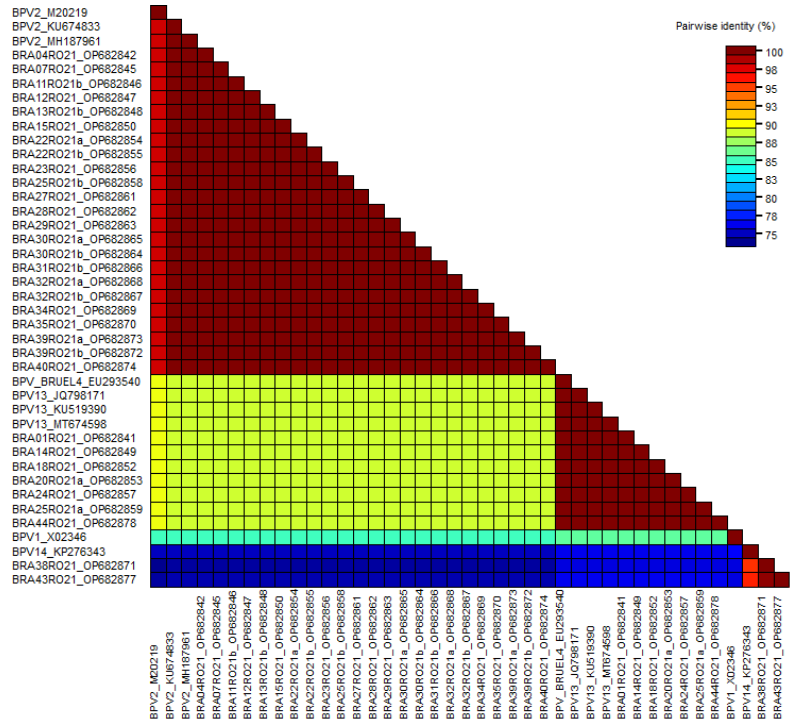

b

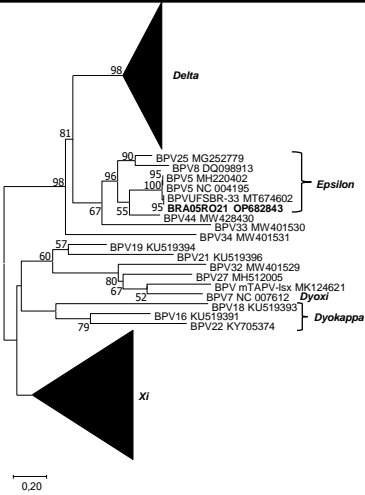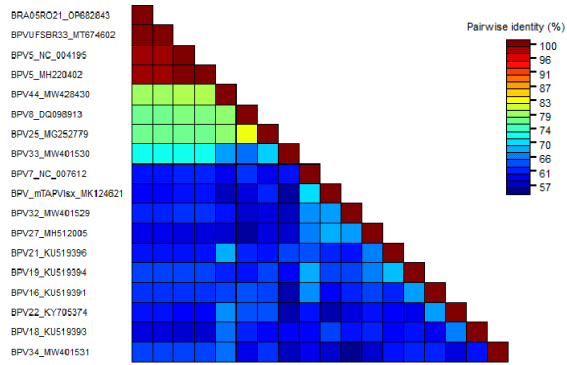

c

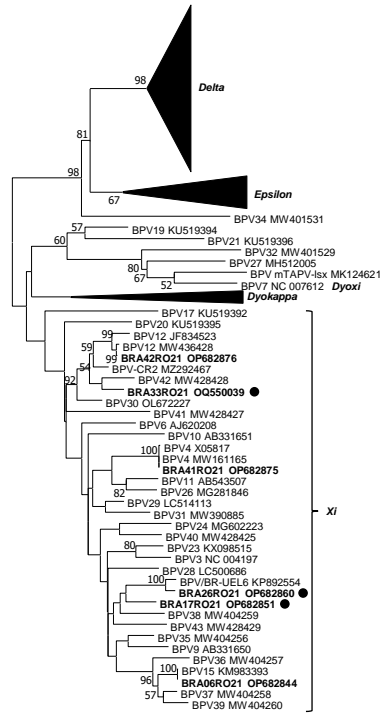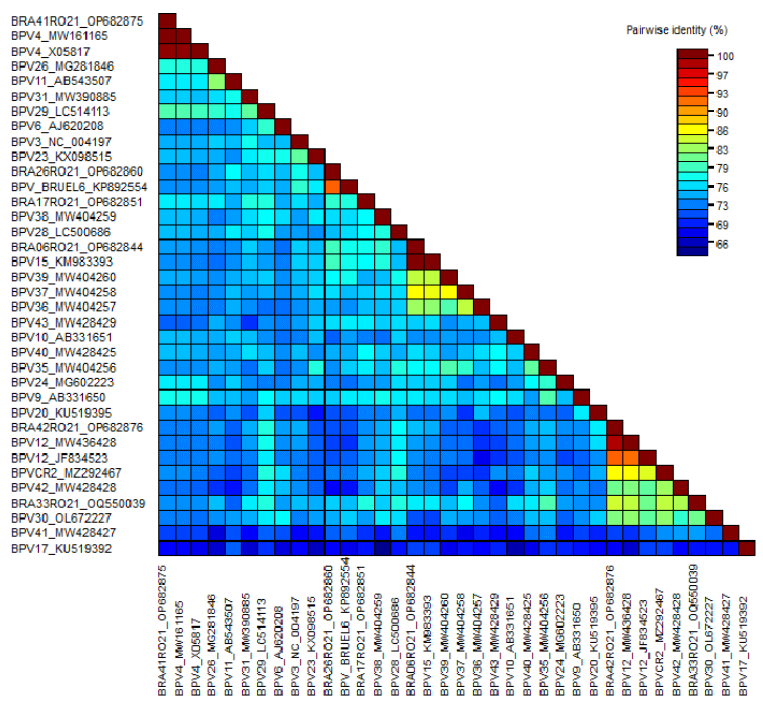

Supplement: Supplementary file 1 [file viruses-17-00719-s001.zip › Supplementary materials Figure S2.pdf]
